# Renin-angiotensin system blockers and COVID-19

**DOI:** 10.1186/s12916-021-02012-6

**Published:** 2021-06-04

**Authors:** Emmanuelle Vidal-Petiot, Nathalie Gault

**Affiliations:** 1 Assistance Publique-Hôpitaux de Paris, Physiology department, Bichat-Claude Bernard University Hospital, 46 rue Henri Huchard, Paris, France; 2 Université de Paris, Inserm U1149, 75018 Paris, France; 3 INSERM CIC-EC 1425, hôpital Bichat Claude Bernard, 75018 Paris, France; 4 APHP.Nord, Département Epidémiologie Biostatistiques et Recherche Clinique, Hôpital Bichat, 75018 Paris, France

**Keywords:** SARS-CoV-2, COVID-19, Renin-angiotensin-aldosterone system, RAS blockers, Angiotensin-converting enzyme inhibitors, Angiotensin receptor blockers

## Background

Early in the coronavirus 2019 (COVID-19) pandemic, angiotensin-converting enzyme 2 (ACE2)—the main counter-regulatory enzyme of the classical renin-angiotensin system (RAS)—was identified as the receptor for the severe acute respiratory syndrome coronavirus 2 (SARS-CoV-2). Two contradictory hypotheses emerged in the scientific literature. Some authors warned against the potential deleterious effect of RAS blockers, which had been shown to increase ACE2 expression in some animal models [1], and advocated for the preventive discontinuation of these drugs [2]. In contrast, others argued that RAS blockers may be beneficial against SARS-CoV-2-induced acute lung injury and should even be introduced in patients with COVID-19 [1, 3]. The latter hypothesis relied on experimental murine models of acute lung injury demonstrating the protective role of ACE2, via the anti-inflammatory and anti-fibrotic actions of angiotensin (1–7) after binding to its Mas receptor, and the deleterious role of ACE, via the actions of angiotensin II after binging to its type 1 receptor (AT1-R) [1]. SARS-CoV-2 allegedly downregulates ACE2 and thereby amplifies angiotensin II-mediated injury: RAS blockers, and in particular AT1-R blockers (ARBs), may thus help restore the disrupted ACE2-angiotensin (1–7)/ACE-angiotensin II homeostasis [3].

## Main text

Multiple observational studies were conducted to clarify this controversial issue and showed no significant association between the chronic use of RAS blockers and either the risk to contract an infection or the risk the develop a severe or lethal form of the disease in infected patients, [4] confirming the statements of scientific societies which all took position against the preventive discontinuation of these drugs.

In contrast, most observational studies which analyzed in-hospital exposure to RAS-blockers concluded in favor of a strong protective effect associated with treatment continuation [1, 5]. However, among the myriad of observational studies published on RAS blockers and COVID-19 since the SARS-CoV-2 outbreak, many have suffered from important methodological limitations [6]. In particular, studies based on in-hospital treatment exposure were criticized for being majorly biased [5]. Exposure assignment in these studies generated immortal-time bias (patients have to survive, or be clinically stable, long enough to achieve the exposure) and a strong indication bias. After hospital admission, RAS blockers tend to be continued in healthier patients and discontinued in patients with hypotension, acute kidney injury, or admitted in intensive care unit, hence with severe forms of the disease, the so-called healthy user-sick stopper bias [5]. Authors often disregarded this typical case of reverse causality and concluded that treatment discontinuation caused disease severity, when the causal relationship was the other way around (disease severity caused treatment discontinuation, and benign disease allowed treatment continuation).

Accordingly, two randomized trials did not confirm the protective role of in-hospital RAS blocker continuation [7, 8]. These trials randomized COVID-19 patients previously treated with RAS blockers and admitted to hospital for treatment continuation or discontinuation and found no difference in disease severity or mortality. However, due to size limitation, these interventional studies did not allow separate analyses of ACE inhibitors (ACEIs) and ARBs, although these may be expected to differentially impact the course of the disease [9]. Overall, management of these medications in infected patients, in particular the specific roles of ACEIs versus ARBs, requires further clarification.

Very interestingly, De Abajo et al. attempted to analyze the effect of ACEI/ARB continuation or discontinuation on COVID-19 outcome (time to in-hospital death) from a retrospective analysis of patients hospitalized in seven hospitals of the Madrid region of Spain from March 1st to March 31st, 2020, but after taking several precautions to avoid the above-mentioned methodological biases [10]. The main improvement compared to previous studies was that the authors separated exposure measurement from outcome measurement. RAS-blocker exposure was measured during a 3-day window after admission, with an intention-to-treat analysis—whatever occurred thereafter—and patients who met the outcome or were discharged within the first 3 days of admission were excluded from all analyses. In addition, the authors carefully accounted for potential confounders by using a Cox regression model adjusted for propensity scores of discontinuation and controlled for potential mediators. Thereby, the immortal-time bias equally impacted both groups, and the indication bias was attenuated (drug cessation within 3 days following admission may still be motivated by signs of severity, which would translate into the occurrence of the outcome after day three).

Out of 625 patients with chronic exposure to RAS blockers, 340 (54%) patients discontinued treatment, with similar rates of discontinuation for ARBs and ACEIs. These high discontinuation rates (mostly driven by hemodynamic instability and/or by the medical distrust for these drugs in the early phase of the pandemic) are in the range order of previous studies reporting in-hospital management of RAS blockers, which are reviewed in the supplementary material of the article.

The main result of this study is that the careful methodological approach of De Abajo and colleagues ironed out the spurious protective effect of treatment continuation found in previous studies. The association between ACEI/ARB (analyzed together or separately) discontinuation and mortality was non-significant. Interestingly, ARBs and ACEIs displayed opposite trends, with adjusted hazard ratios (HR) for discontinuation versus continuation of 1.59 (95% CI 0.89–2.85) and 0.70 (95% CI 0.42–1.17) for ARBs and ACEIs, respectively. Patients who were on ARBs and continued treatment appeared to have a better prognosis than patients who were on ACEI and continued treatment, with mortality rates of 20.8 and 33.1% respectively, yielding a fully adjusted HR of 0.52 (95% CI 0.29–0.93). This head-to-head comparison of ACEIs and ARBs has the advantage that groups are submitted to the same prescription bias and raises interesting hypotheses with potential therapeutic impact. However, these retrospective observational data need to be interpreted with extreme caution until the results of ongoing trials randomizing patients hospitalized for COVID-19 to receive an ARB or placebo are published [3].

## Conclusions

In summary, unlike previously reported in studies suffering methodological flaws, and in line with recently published small-scale randomized trials, there is no strong protective effect associated with the continuation of RAS blockers after hospital admission for COVID-19. The potential superiority of ARBs versus ACEIs is all the more interesting as it is supported by a pathophysiological rationale, but warrants confirmation by randomized trials.

## Data Availability

Not applicable
